# ECT-induced primary open-angle glaucoma in a patient with unstable thyroid function: a case report

**DOI:** 10.3389/fpsyt.2025.1497205

**Published:** 2025-03-05

**Authors:** Cuiyuan Fu, Xiuzhen Yang, Kun Li

**Affiliations:** ^1^ Department of Physical Therapy, Shandong Daizhuang Hospital, Jining, China; ^2^ Jining Key Laboratory of Neuromodulation, Jining, China

**Keywords:** Schizophrenia, Hyperthyroidism, Glaucoma, electroconvulsive therapy, safety, effectivity

## Abstract

**Background:**

Schizophrenia is a prevalent and severe psychiatric disorder for which electroconvulsive therapy (ECT) is frequently utilized as a treatment modality. Although ECT can transiently elevate intraocular pressure, the incidence of ECT-related adverse ophthalmic events in patients with coexisting hyperthyroidism is not well documented.

**Case report:**

In this report, we describe an elderly woman with schizophrenia and hyperthyroidism. Before undergoing ECT, she had no previous history of glaucoma, and her thyroid function was in an unstable state. After three sessions of ECT, the patient exhibited conjunctival congestion and was subsequently diagnosed with primary open-angle glaucoma, which was not treated. Her intraocular pressure normalized prior to and following the fourth ECT session, and she experienced no further ocular discomfort during subsequent treatments (fifth through eighth sessions).

**Conclusions:**

Although ECT has been used in patients with coexisting psychiatric and thyroid dysfunction, there is a lack of reports addressing the risk of inducing or exacerbating glaucoma in the context of unstable thyroid function. This case emphasizes the necessity of monitoring intraocular pressure in patients with unstable thyroid function during ECT, to mitigate the risk of ocular complications.

## Introduction

Schizophrenia is a disabling condition affecting approximately 1% of the global population (1). According to the Global Burden of Disease Study 2019 report, the prevalence, incidence, and disability-adjusted life years associated with schizophrenia have increased by 65%, 37%, and 65%, respectively (2). Among older adults with schizophrenia, particularly those who have not responded adequately to pharmacological treatments, electroconvulsive therapy (ECT) is emerging as an effective therapeutic option (3). ECT, a neuromodulatory intervention with a well-established safety profile, can markedly improve both the positive and negative symptoms of schizophrenia (4).

Given that schizophrenia often requires long-term management and that elderly patients may have multiple comorbidities, understanding the safety profile of ECT in this population is crucial. Notably, the prevalence of glaucoma increases with age, making older adults more susceptible to this condition. Aging is associated with various structural and functional changes within the eye, including reduced outflow of aqueous humor, leading to elevated intraocular pressure (IOP) and a higher risk of glaucoma (5).

Glaucoma remains a leading cause of irreversible vision loss worldwide, and elevated IOP identified as a principal risk factor (5). ECT transiently increase IOP, potentially contributing to glaucoma risk due to the use of muscle relaxants, anesthetic agents, and seizure induction (6–8). While individuals without pre-existing ocular conditions generally do not experience clinically significant elevations in IOP during ECT, patients with glaucoma may be more vulnerable (9). However, there are case reports indicating that, IOP elevation is less pronounced in patients with glaucoma (10), and IOP may even decrease following ECT (11).

Additionally, individuals with unstable thyroid function are at a higher risk of developing glaucoma (12). However, earlier reports of ECT in patients with comorbid psychiatric disorders and hyperthyroidism—whether stable or unstable—have not documented ocular complications (13, 14). Therefore, the interplay between unstable thyroid function and ECT-induced glaucoma remains unclear.

In view of these uncertainties, further evidence is needed to clarify whether ECT can precipitate or worsen glaucoma in individuals with unstable thyroid function. Here, we present a case that suggests a potential link between uncontrolled thyroid status and the emergence of glaucoma during the course of ECT treatment, highlighting the need for more careful monitoring and assessment in these patients.

## Case description

The patient is a 60-year-old female who was admitted to our hospital under the compulsion of her family. She has a 13-year history of schizophrenia, which progressively worsened following the death of her mother. Her symptoms included auditory hallucinations and paranoid delusions, with the patient believing she was being targeted for harm. She was diagnosed with schizophrenia and initially responded well to clozapine treatment. Three years prior to admission, a phone call from her sister-in-law triggered a relapse, intensifying her paranoid delusions about family disturbances. She has experienced persistent auditory hallucinations and sometimes has delusions of ruin, such as the idea of “the earth exploding”.

The auxiliary examination conducted did not reveal any significant changes, thus excluding organic damage and physical illnesses. During her most recent admission, she presented with primary symptoms of auditory hallucinations, persecutory delusions, emotional instability, and refusal to eat, persisting for three days. Three days before admission, she attended a class reunion and became emotionally unstable after being informed that her former teacher had a heart condition and no one to care for her. After returning home, she grew agitated and she insisted her classmates visit the teacher by repeatedly calling them. Meanwhile, she frequently broke down crying, deeply troubled by the news. Her symptoms included auditory hallucinations, arguing with perceived insults, paranoid delusions fearing harm from others, social withdrawal, delusions of ruin (e.g., “I deserve to die” and “The world is ending”), refusal to eat, and passive engagement with her surroundings. In addition to observing a disruption in the sleep-wake cycle, which further attested to the severity of her illness, the patient was frequently found to be staring blankly and unresponsive to questions.

### Past medical, personal, and family history

The patient was diagnosed with hyperthyroidism 10 years ago and has been maintained on long-term methimazole treatment (2.5 mg daily) with regular thyroid function tests within normal ranges. The patient has no history of glaucoma. Neither the patient nor her family reported any significant past physical illnesses. There was no history of smoking, alcohol use, or substance abuse. Both the patient and her family denied any history of mental illness in the family.

### Physical, mental and laboratory examinations

Upon admission, the patient’s vital signs were: temperature 36.8°C, pulse 84 bpm, respiration 20 breaths/min, and blood pressure 136/82 mmHg. Cardiovascular, respiratory, and digestive system examinations showed no significant abnormalities. Neurological examination revealed normal gross vision, normal visual fields, clear optic disc margins, no retinal hemorrhages or exudates, normal eye movements, no nystagmus, bilaterally equal and reactive pupils (3 mm), and intact direct and consensual light reflexes.

The mental status examination revealed the following findings: the patient exhibited passive admission, with a tense facial expression and passive interaction. She was capable of self-care in daily life but showed a lack of initiative, often responding to questions with silence or vague answers. She was unresponsive to repeated inquiries from medical staff and family members, demonstrating clear consciousness but a profound lack of insight into her condition. Auditory hallucinations, persecutory and ruin delusions were elicited. Regarding mood, there was no evidence of depressive syndrome, anxiety syndrome, or manic syndrome. However, her volition and behavior were clearly affected by passivity, which is consistent with catatonic features. There was no pathological enhancement or reduction in volition or behavior, but the patient’s passive engagement with her environment suggests a disruption in normal volitional function. Additionally, a disruption in the sleep-wake cycle was noted, further indicating the severity of her illness.

Laboratory results showed normal ranges for complete blood count, stool, urine, liver function, renal function, electrolytes, blood glucose, and parathyroid hormone. Electrocardiogram and chest CT were unremarkable. Thyroid function tests showed thyroid-stimulating hormone (TSH) 0.005 mU/L (reference range, 0.27-4.2 mU/L), triiodothyronine (T3) 6.17 nmol/L (reference range, 1.3-3.1 nmol/L), free triiodothyronine (FT3) 22.82 pmol/L (reference range, 3.6-7.5 pmol/L), thyroxine (T4) 296.00 nmol/L (reference range, 62-164 nmol/L), free thyroxine (FT4) 77.56 pmol/L (reference range, 12.0-22.0 pmol/L). Thyroid ultrasound revealed a hypoechoic nodule in the lower right lobe and an unevenly hypoechoic area in the posterior right lobe, potentially originating from the parathyroid gland. Thyroid antibodies were: thyroglobulin 0.2 μg/L (reference range, 1.4-78 μg/L), anti-thyroglobulin antibody 2989.00 IU/ml (reference range, <115 IU/ml), anti-thyroid peroxidase antibody 338.50 IU/ml (reference range, <34 IU/ml), and TSH receptor antibody 28.17 IU/L (reference range, <3 IU/L).

### Diagnosis

The patient’s clinical presentation and history meet the diagnostic criteria for schizophrenia (code F20.9), as outlined in the Diagnostic and Statistical Manual of Mental Disorders, 5th edition (DSM-5). She exhibits prominent delusions, auditory hallucinations, and significant impairment in social and occupational functioning, persisting for more than six months. Additionally, her presentation includes features consistent with catatonia, such as passive behavior, unresponsiveness, and lack of initiative, which align with the International Classification of Diseases, 10th edition (ICD-10) criteria for catatonic schizophrenia (code F20.2).

### Treatment course

Upon admission, in consideration of the obvious auditory hallucinations, paranoid and ruin delusions, as well as poor treatment compliance and passive behavior, the patient was started on intravenous fluids (1500 ml/day), risperidone 1 mg twice daily, and clozapine 50 mg nightly. Two days after admission, the patient’s symptoms, such as auditory hallucinations and delusions, remained severe. On Day 3, the use of modified ECT placed bilaterally at the temporal regions was initiated (see **Table 1**), and the dose of clozapine was increased to 75 mg daily. The second ECT session was conducted on Day 4, followed by the third session on Day 5. On Day 7, the dose of risperidone was increased to 2 mg twice daily. The fourth ECT session was administered on Day 9, the fifth on Day 11, and the sixth on Day 13. On Day 14, the dose of risperidone was gradually titrated to 5 mg daily. The seventh ECT session was performed on Day 19, and the dose of clozapine was increased to 125 mg daily. The eighth ECT session was conducted on Day 23, and by Day 30, the dose of clozapine was further increased to 150 mg daily. Based on thyroid function test results upon admission, the endocrinologist recommended gradually increasing the methimazole dosage to 20 mg per day.

**Table 1:** Treatment parameters of electroconvulsive therapy and intraocular pressure monitoring.

Session	Sodium thiopental (mg)	Suxamethonium chloride (mg)	Electric time (s)	Energy (J)	Current (mA)	Tetanus	Clonus	Intraocular pressure (mmHg)
1	120	30	3.5	45.3	700	+	+	–
2	120	30	3.5	45.3	700	+	+	–
3	120	30	3.5	45.3	700	+	+	After ECT: R-12.1, L-26.8
4	120	30	3.5	45.3	900	+	+	Before ECT: R-10.0, L-11.7
								After ECT: R-11.0, L-13.5
5	120	30	3.5	45.3	900	+	+	–
6	120	30	3.5	45.3	900	+	+	–
7	120	30	3.5	45.3	900	+	+	–
8	120	30	3.5	45.3	900	+	+	–

R, right eye; L, left eye; ECT, electroconvulsive therapy.

After the third ECT session, the patient still experienced auditory hallucinations, paranoid and ruin delusions but began to eat and engage in activities independently, leading to the discontinuation of intravenous fluids. She developed bilateral conjunctival hyperemia and edema without discomfort or significant vision loss. An ophthalmologist consultation and non-contact tonometry revealed IOP of 12.1 mmHg in the right eye and 26.8 mmHg in the left eye, with delayed P100 latency in visual evoked potentials, suggestive of primary open-angle glaucoma. No treatment was administered, and the condition was monitored.

Before the fourth ECT session, IOP measurements were 10.0 mmHg in the right eye and 11.7 mmHg in the left eye, and one hour after treatment, they were 11.0 mmHg and 13.5 mmHg, respectively, with no recurrence of conjunctival hyperemia or edema.

**Figure 1:**
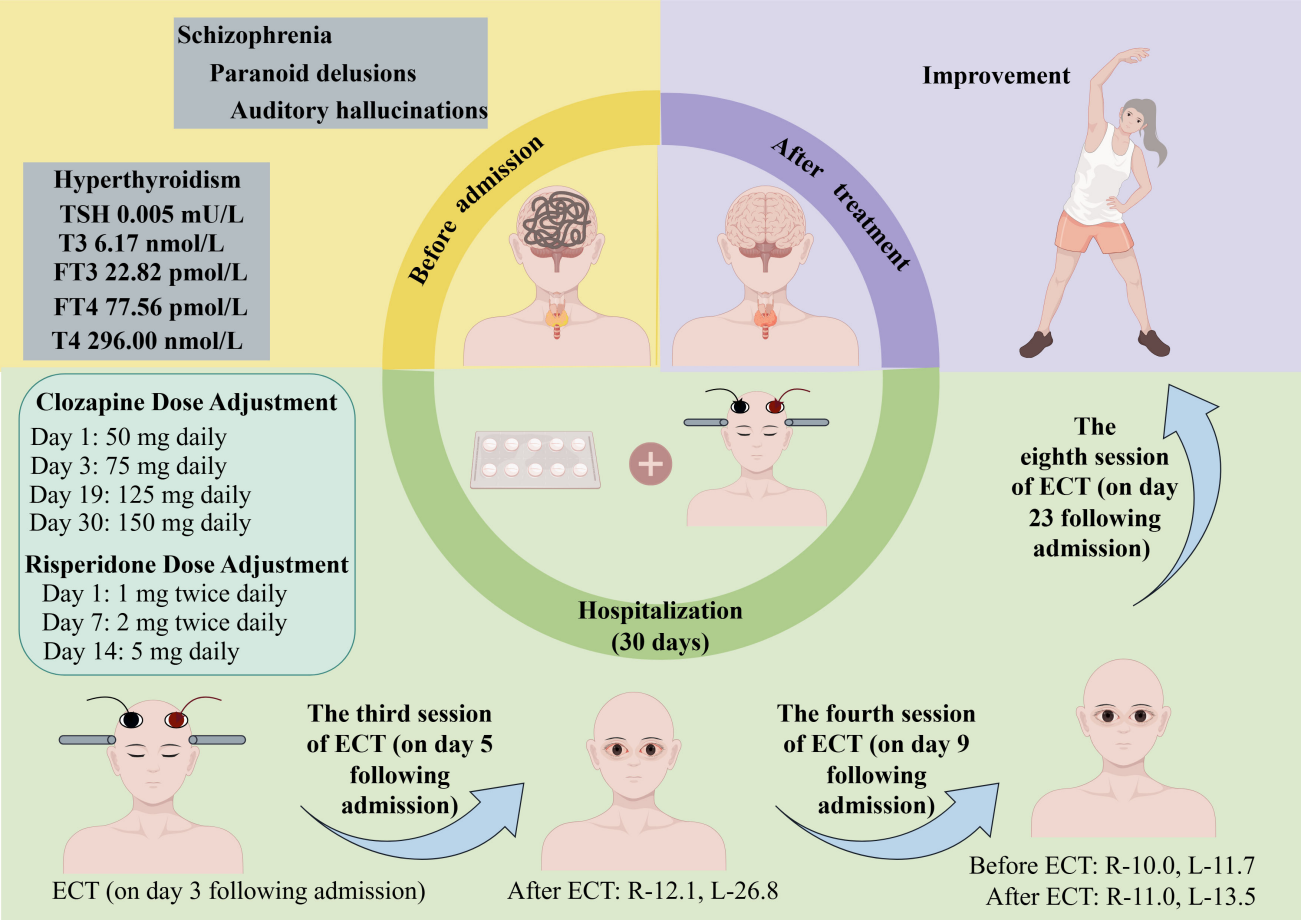
Case summary. R-12.1 indicates that the intraocular pressure of the right eye is 12.1 mmHg; R-10.0, the intraocular pressure of the right eye is 10.0 mmHg; R-11.0, the intraocular pressure of the right eye is 11.0 mmHg; L-26.8, the intraocular pressure of the left eye is 26.8 mmHg; L-11.7, the intraocular pressure of the left eye is 11.7 mmHg; L-13.5, the intraocular pressure of the left eye is 13.5 mmHg. ECT, electroconvulsive therapy; FT3, free triiodothyronine; FT4, free thyroxine; TSH, thyroid-stimulating hormone; T3, triiodothyronine; T4, thyroxine.

From the fifth to the eighth ECT sessions, the patient reported no ocular discomfort. Following eight ECT treatments, the patient’s auditory hallucinations, paranoid and ruin delusions resolved, her mood stabilized, she ate well, and engaged actively with others, although she had some short-term memory impairment, unable to accurately recall breakfast details. The patient’s condition showed significant improvement (see **Figure 1**).

## Discussion

In the early 20th century, a theory emerged that ECT was prone to causing high intraocular pressure and presented a risk to glaucoma patients (15). The relevant influencing factors include the muscle relaxants and anesthetics used during ECT, the epileptic seizures induced by ECT, and individual differences (6–9).

In current research, there are studies examining the impact of ECT on thyroid function and sex hormones in patients with depressive episodes, finding that ECT may have a certain effect on sex hormone levels, but its impact on thyroid function is not significant (16). Additionally, research progress on the neurobiological mechanisms of ECT treatment has indicated that ECT involves various neurophysiological and neurochemical changes, but there is currently no direct evidence to suggest that ECT affects thyroid function (17). In our case report, the patient’s thyroid function was unstable before ECT. Her thyroid hormone levels indicated hyperthyroidism. After the third ECT session, her intraocular pressure increased and was accompanied by conjunctival congestion and swelling. Taking into account the potential influence of thyroid dysfunction on a patient’s physiological and biochemical responses, there could be an associated risk of glaucoma induced by ECT. The disruption of thyroid hormones in the patient may also be associated with the use of clozapine, as clozapine has the potential to alter the normal feedback mechanism of thyroid hormones (18). However, some studies have reported that clozapine does not have an effect on thyroid hormone levels (19).

Can schizophrenia with comorbid hyperthyroidism be treated with ECT? As reported by Oglodek et al. (20), the ECT performed after stabilizing thyroid function can effectively ameliorate psychiatric symptoms. However, there is also a report indicating that during periods of unstable thyroid function in patients, the ECT can also effectively improve psychiatric symptoms, with no adverse events occurring (14).

The improvement of psychiatric disorders comorbid with hyperthyroidism can be attributed to two primary mechanisms. Firstly, the ECT can normalize the levels of certain proteins whose expression is altered by T3-receptor complexes, as genes typically have multiple upstream regulatory sites (21). Secondly, some cellular responses induced by ECT can mitigate the pathological reactions caused by hyperthyroidism (22). Thus, the ECT can be conducted in the presence of thyroid hormone imbalance, but it is also necessary to monitor the patient’s intraocular pressure.

In our case report, the patient had no history of glaucoma prior to receiving ECT. During the course of ECT, after experiencing ocular discomfort, upon consultation with an ophthalmologist, she was diagnosed with primary open-angle glaucoma. However, instead of treating it, we chose to observe her intraocular pressure. It returned to normal levels before and after the fourth ECT session, and no further ocular discomfort was reported after the fifth to eighth ECT sessions. This case report serves as a reminder that we need to be aware of the risk of ECT inducing glaucoma in patients with hyperthyroidism.

## Limitation

The patient’s thyroid function indicators were not re-examined, so we cannot specifically determine what impact ECT has had on the hyperthyroidism indicators. This is something we need to pay attention to in our future clinical work.

## Conclusion

Patients with schizophrenia accompanied by hyperthyroidism can use ECT to improve psychiatric symptoms, the ECT poses a risk of inducing glaucoma for patients with unstable thyroid function. There is currently a lack of reports on the negative impact of ECT on patients with unstable thyroid function, and our case report complements this aspect, it is necessary to closely monitor the patient’s intraocular pressure if they are in a state of unstable thyroid function.

## Data Availability

The original contributions presented in the study are included in the article/**Supplementary Material**. Further inquiries can be directed to the corresponding author/s.
